# *trans*-Tetra­iodidobis(tri-*p*-tolyl­phosphine oxide-κ*O*)tin(IV)

**DOI:** 10.1107/S1600536808023945

**Published:** 2008-08-06

**Authors:** Marina Hitosugi-Levesque, Joseph M. Tanski

**Affiliations:** a Department of Chemistry, Vassar College, Poughkeepsie, NY 12604, USA

## Abstract

The centrosymmetric title compound, [SnI_4_(C_21_H_21_OP)_2_], is a monomeric complex that displays a nearly octa­hedral coordination of tin(IV), with an Sn—O bond distance of 2.159 (2) Å and an average Sn—I bond distance of 2.79 (3) Å.

## Related literature

For examples of structurally characterized tin(IV) halide complexes of phosphine oxide ligands, see: Tursina *et al.* (1985 ▶); Tursina, Aslanov *et al.* (1986 ▶); Tursina, Yatsenko *et al.* (1986 ▶); Tudela *et al.* (1993 ▶); Genge *et al.* (1999 ▶); Szymanska-Buzar *et al.* (2001 ▶); Davis, Clarke *et al.* (2006 ▶); Davis, Levason *et al.* (2006 ▶); Caldwell & Tanski (2008 ▶). For related literature, see: Levason *et al.* (2003 ▶); Woollins (2003 ▶).
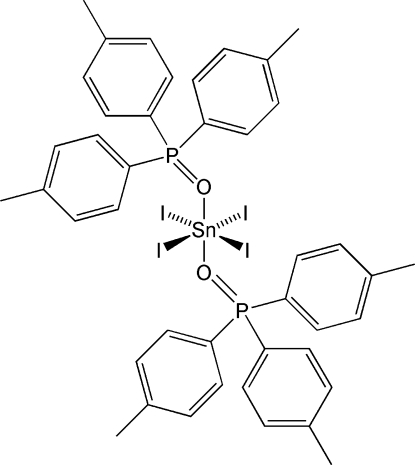


## Experimental

### 

#### Crystal data

[SnI_4_(C_21_H_21_OP)_2_]
*M* *_r_* = 1266.99
Orthorhombic, 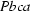

*a* = 18.6013 (11) Å
*b* = 11.9677 (7) Å
*c* = 19.3335 (11) Å
*V* = 4303.9 (4) Å^3^
*Z* = 4
Mo *K*α radiation
μ = 3.57 mm^−1^
*T* = 125 (2) K
0.20 × 0.20 × 0.05 mm

#### Data collection

Bruker APEXII CCD diffractometer
Absorption correction: multi-scan (*SADABS*; Bruker, 2007 ▶) *T*_min_ = 0.535, *T*_max_ = 0.842
53839 measured reflections
5331 independent reflections
4841 reflections with *I* > 2σ(*I*)
*R*_int_ = 0.032

#### Refinement

*R*[*F* ^2^ > 2σ(*F* ^2^)] = 0.019
*wR*(*F* ^2^) = 0.048
*S* = 1.07
5331 reflections
235 parameters
H-atom parameters constrained
Δρ_max_ = 0.56 e Å^−3^
Δρ_min_ = −0.49 e Å^−3^

### 

Data collection: *APEX2* (Bruker, 2007 ▶); cell refinement: *SAINT* (Bruker, 2007 ▶); data reduction: *SAINT*; program(s) used to solve structure: *SHELXS97* (Sheldrick, 2008 ▶); program(s) used to refine structure: *SHELXL97* (Sheldrick, 2008 ▶); molecular graphics: *SHELXTL* (Sheldrick, 2008 ▶); software used to prepare material for publication: *SHELXTL*.

## Supplementary Material

Crystal structure: contains datablocks global, I. DOI: 10.1107/S1600536808023945/pv2089sup1.cif

Structure factors: contains datablocks I. DOI: 10.1107/S1600536808023945/pv2089Isup2.hkl

Additional supplementary materials:  crystallographic information; 3D view; checkCIF report

## Figures and Tables

**Table:** 

Sn—O1	2.1590 (15)
Sn—I1	2.76735 (17)
Sn—I2	2.8158 (2)
O1—P1	1.5207 (16)

**Table:** 

O1—Sn—O1^i^	180
O1—Sn—I1	89.84 (4)
O1^i^—Sn—I1	90.16 (4)
I1—Sn—I1^i^	180
O1—Sn—I2^i^	93.59 (4)
O1^i^—Sn—I2^i^	86.41 (4)

Symmetry code: (i) 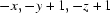.

**Fig. 1.**
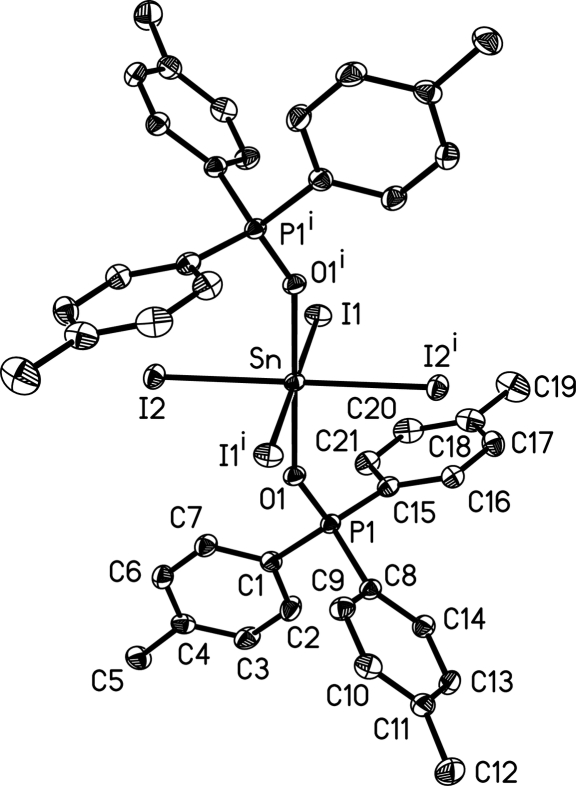
A view of complex (I) with displacement ellipsoids shown at the 50% probability level. H atoms have been omitted for clarity. Symmetry code: i = -x, -y+1, -z+1

## supplementary crystallographic information

### Comment

Tin(IV) iodide may be readily prepared by oxidation of tin metal with iodine (Woollins, 2003). A relatively weak Lewis acid, SnI_4_ nevertheless forms complexes with phosphines and phosphine oxides (Genge *et al.* 1999; Davis, Clarke *et al.* 2006; Caldwell & Tanski, 2008). The phosphine oxide complexes are chiefly obtained by air oxidation of the phosphine ligands in the presence of the tin(IV) halide (Levason *et al.* 2003). The crystal and molecular structures of the bis(triphenyl phosphine oxide) adducts of Sn*X*_4_, where *X* = F, Cl, Br and I, have all been previously reported. In the case of the fluoride, *trans*-[Ph_3_PO]_2_SnF_4_, the the phospine oxides are mutually *trans* (Davis, Clarke *et al.* 2006). The chloride, *cis*-[Ph_3_PO]_2_SnCl_4_, exhibits *cis* phospine oxides ligands (Tursina *et al.* 1985; Szymanska-Buzar *et al.* 2001), although it has been reported that both the *cis* and *trans* isomers are observed in solution by ^31^P NMR (Davis, Levason *et al.* 2006<). The structures of both *cis*-[Ph_3_PO]_2_SnBr_4_ and *trans*-[Ph_3_PO]_2_SnBr_4_ are known for the bromide (Tudela *et al.* 1993; Tursina, Yatsenko *et al.* 1986). In the structure of the iodide, the triphenyl phosphine oxide ligands of *cis*-[Ph_3_PO]_2_SnI_4_ are found to be *cis* (Tursina, Aslanov *et al.* 1986). As reported here, tri(*p*-tolyl) phosphine oxide results in an iodide complex wherein the phosphine oxide ligands are found to be *trans*.

Reaction of SnI_4_ with tri(*p*-tolyl) phosphine, (*p*-CH_3_C_6_H_4_)_3_P, in CHCl_3_ in the presence of air afforded the title complex [(*p*-CH_3_C_6_H_4_)_3_PO]_2_SnI_4_, (I).

Complex (I) exhibits a nearly octahedral coordination at tin, which resides on a crystallographic inversion center. The phosphine oxide ligands are mutually *trans*, with an Sn—O distance of 2.159 (2) Å, and Sn—I distances of 2.7674 (2) and 2.8158 (2) Å. Relevant bond lengths and angles can be found in Table 1. Despite the *p*-CH_3_ substituent and *trans* orientation of the phosphine oxide ligands in (I), the Sn—O and Sn—I distances in (I) are very similar to those found in *cis*-[Ph_3_PO]_2_SnI_4_, wherein the Sn—O distances are 2.15 (2) and 2.11 (2) Å, and the Sn—I distances range from 2.781 (2) to 2.816 (2) Å (Tursina, Aslanov *et al.* 1986).

### Experimental

Complex (I) was prepared by treating a chloroform (*ca* 10 ml) solution of SnI_4_ (626 mg, 1.00 mmol) with an excess of (*p*-CH_3_C_6_H_4_)_3_P (647 mg, 2.13 mmol) in the presence of air. Suitable crystals for single-crystal X-ray analysis separated as orange plates within 2 weeks at room temperature.

### Refinement

H atoms on carbon atoms were included in calculated positions using a riding model at C—H distances 0.95 and 0.98 Å and U_iso_(H) = 1.2 and 1.5U_eq_(C) of the aryl and methyl C-atoms, respectively.

### Crystal data

**Table:**

[SnI_4_(C_21_H_21_OP)_2_]	*F*_000_ = 2408
*M**_r_* = 1266.99	*D*_x_ = 1.955 Mg m^−^^3^
Orthorhombic, *P**b**c**a*	Mo *K*α radiation λ = 0.71073 Å
Hall symbol: -P2ac2ab	Cell parameters from 9901 reflections
*a* = 18.6013 (11) Å	θ = 2.8–28.3º
*b* = 11.9677 (7) Å	µ = 3.57 mm^−^^1^
*c* = 19.3335 (11) Å	*T* = 125 (2) K
*V* = 4303.9 (4) Å^3^	Plate, orange
*Z* = 4	0.20 × 0.20 × 0.05 mm

### Data collection

**Table:**

Bruker APEXII CCD diffractometer	5331 independent reflections
Radiation source: fine-focus sealed tube	4841 reflections with *I* > 2σ(*I*)
Monochromator: graphite	*R*_int_ = 0.032
*T* = 125(2) K	θ_max_ = 28.3º
φ and ω scans	θ_min_ = 2.1º
Absorption correction: multi-scan(SADABS; Bruker, 2007)	*h* = −24→24
*T*_min_ = 0.535, *T*_max_ = 0.842	*k* = −15→15
53839 measured reflections	*l* = −25→25

### Refinement

**Table:**

Refinement on *F*^2^	Secondary atom site location: difference Fourier map
Least-squares matrix: full	Hydrogen site location: inferred from neighbouring sites
*R*[*F*^2^ > 2σ(*F*^2^)] = 0.019	H-atom parameters constrained
*wR*(*F*^2^) = 0.048	*w* = 1/[σ^2^(*F*_o_^2^) + (0.0232*P*)^2^ + 4.4313*P*] where *P* = (*F*_o_^2^ + 2*F*_c_^2^)/3
*S* = 1.07	(Δ/σ)_max_ = 0.001
5331 reflections	Δρ_max_ = 0.56 e Å^−^^3^
235 parameters	Δρ_min_ = −0.49 e Å^−^^3^
Primary atom site location: structure-invariant direct methods	Extinction correction: none

### Special details

**Table:**

Geometry. All e.s.d.'s (except the e.s.d. in the dihedral angle between two l.s. planes) are estimated using the full covariance matrix. The cell e.s.d.'s are taken into account individually in the estimation of e.s.d.'s in distances, angles and torsion angles; correlations between e.s.d.'s in cell parameters are only used when they are defined by crystal symmetry. An approximate (isotropic) treatment of cell e.s.d.'s is used for estimating e.s.d.'s involving l.s. planes.
Refinement. A suitable crystal was mounted in a nylon loop with Paratone-*N* cryoprotectant oil and data was collected on a Bruker *APEX* 2 CCD platform diffractometer. The structure was solved using direct methods and standard difference map techniques, and was refined by full-matrix least-squares procedures on *F*^2^ with *SHELXTL* Version 6.14 (Sheldrick, 2008). All non-hydrogen atoms were refined anisotropically. Refinement of *F*^2^ against ALL reflections. Refinement of *F*^2^ against ALL reflections. The weighted *R*-factor *wR* and goodness of fit *S* are based on *F*^2^, conventional *R*-factors *R* are based on *F*, with *F* set to zero for negative *F*^2^. The threshold expression of *F*^2^ > σ(*F*^2^) is used only for calculating *R*-factors(gt) *etc*. and is not relevant to the choice of reflections for refinement. *R*-factors based on *F*^2^ are statistically about twice as large as those based on *F*, and *R*-factors based on ALL data will be even larger. EXTI refined to zero and was removed from the refinement.

### Fractional atomic coordinates and isotropic or equivalent isotropic displacement parameters (Å^2^)

**Table:**

	*x*	*y*	*z*	*U*_iso_*/*U*_eq_	
Sn	0.0000	0.5000	0.5000	0.01453 (5)	
I1	0.071249 (8)	0.378968 (13)	0.399124 (8)	0.02266 (4)	
I2	0.042286 (8)	0.699279 (12)	0.434117 (8)	0.02186 (4)	
O1	−0.09443 (8)	0.50578 (13)	0.43516 (8)	0.0188 (3)	
P1	−0.15379 (3)	0.44482 (5)	0.39642 (3)	0.01603 (11)	
C1	−0.17934 (12)	0.52832 (19)	0.32327 (11)	0.0194 (4)	
C2	−0.23667 (13)	0.4945 (2)	0.28165 (13)	0.0265 (5)	
H2A	−0.2605	0.4259	0.2910	0.032*	
C3	−0.25902 (13)	0.5601 (2)	0.22692 (13)	0.0266 (5)	
H3A	−0.2976	0.5355	0.1985	0.032*	
C4	−0.22576 (12)	0.6619 (2)	0.21282 (12)	0.0221 (5)	
C5	−0.25292 (14)	0.7344 (2)	0.15496 (13)	0.0291 (5)	
H5A	−0.2205	0.7982	0.1488	0.044*	
H5B	−0.3012	0.7615	0.1663	0.044*	
H5C	−0.2547	0.6908	0.1121	0.044*	
C6	−0.16762 (13)	0.6945 (2)	0.25375 (13)	0.0242 (5)	
H6A	−0.1433	0.7624	0.2438	0.029*	
C7	−0.14473 (13)	0.6286 (2)	0.30905 (12)	0.0222 (5)	
H7A	−0.1055	0.6522	0.3369	0.027*	
C8	−0.23153 (12)	0.43203 (19)	0.45048 (11)	0.0181 (4)	
C9	−0.24193 (13)	0.5130 (2)	0.50135 (12)	0.0224 (5)	
H9A	−0.2060	0.5679	0.5092	0.027*	
C10	−0.30407 (13)	0.5139 (2)	0.54033 (13)	0.0245 (5)	
H10A	−0.3103	0.5696	0.5748	0.029*	
C11	−0.35776 (13)	0.4346 (2)	0.53003 (13)	0.0242 (5)	
C12	−0.42613 (15)	0.4397 (2)	0.57128 (16)	0.0351 (6)	
H12A	−0.4445	0.5165	0.5713	0.053*	
H12B	−0.4166	0.4161	0.6189	0.053*	
H12C	−0.4620	0.3899	0.5505	0.053*	
C13	−0.34695 (13)	0.3523 (2)	0.47927 (13)	0.0238 (5)	
H13A	−0.3827	0.2970	0.4719	0.029*	
C14	−0.28479 (12)	0.3509 (2)	0.43988 (12)	0.0209 (4)	
H14A	−0.2782	0.2948	0.4056	0.025*	
C15	−0.12942 (12)	0.31033 (19)	0.36393 (11)	0.0181 (4)	
C16	−0.13571 (12)	0.21535 (19)	0.40494 (12)	0.0204 (4)	
H16A	−0.1573	0.2205	0.4494	0.024*	
C17	−0.11054 (14)	0.1132 (2)	0.38123 (13)	0.0246 (5)	
H17A	−0.1159	0.0486	0.4094	0.029*	
C18	−0.07758 (14)	0.1039 (2)	0.31675 (13)	0.0275 (5)	
C19	−0.05059 (18)	−0.0075 (3)	0.29219 (16)	0.0406 (7)	
H19A	−0.0021	0.0011	0.2731	0.061*	
H19B	−0.0828	−0.0365	0.2563	0.061*	
H19C	−0.0492	−0.0599	0.3311	0.061*	
C20	−0.07179 (14)	0.1986 (2)	0.27588 (13)	0.0293 (5)	
H20A	−0.0499	0.1932	0.2316	0.035*	
C21	−0.09738 (13)	0.3010 (2)	0.29838 (12)	0.0249 (5)	
H21A	−0.0933	0.3649	0.2695	0.030*	

### Atomic displacement parameters (Å^2^)

**Table:**

	*U*^11^	*U*^22^	*U*^33^	*U*^12^	*U*^13^	*U*^23^
Sn	0.01273 (9)	0.01808 (10)	0.01278 (9)	−0.00027 (7)	0.00002 (7)	0.00045 (7)
I1	0.02116 (8)	0.02856 (9)	0.01826 (7)	0.00429 (6)	0.00170 (5)	−0.00372 (6)
I2	0.02329 (8)	0.02232 (8)	0.01999 (8)	−0.00454 (6)	0.00017 (6)	0.00311 (6)
O1	0.0151 (7)	0.0217 (8)	0.0195 (8)	−0.0017 (6)	−0.0029 (6)	0.0009 (6)
P1	0.0144 (2)	0.0187 (3)	0.0150 (3)	−0.0020 (2)	−0.0015 (2)	0.0013 (2)
C1	0.0182 (10)	0.0223 (11)	0.0177 (10)	−0.0002 (9)	−0.0020 (8)	0.0027 (8)
C2	0.0247 (12)	0.0271 (12)	0.0276 (12)	−0.0088 (10)	−0.0078 (10)	0.0076 (10)
C3	0.0203 (11)	0.0366 (14)	0.0229 (12)	−0.0047 (10)	−0.0068 (9)	0.0041 (10)
C4	0.0193 (10)	0.0288 (12)	0.0182 (11)	0.0042 (9)	0.0010 (9)	0.0037 (9)
C5	0.0272 (12)	0.0343 (14)	0.0259 (12)	0.0038 (11)	−0.0023 (10)	0.0100 (11)
C6	0.0237 (11)	0.0236 (11)	0.0253 (12)	−0.0030 (9)	0.0001 (9)	0.0056 (9)
C7	0.0202 (11)	0.0246 (12)	0.0216 (11)	−0.0041 (9)	−0.0039 (9)	0.0009 (9)
C8	0.0158 (10)	0.0217 (11)	0.0169 (10)	−0.0011 (8)	−0.0010 (8)	0.0022 (8)
C9	0.0197 (11)	0.0244 (11)	0.0231 (11)	−0.0022 (9)	−0.0029 (9)	−0.0030 (9)
C10	0.0249 (11)	0.0270 (12)	0.0215 (11)	0.0029 (10)	0.0007 (9)	−0.0019 (9)
C11	0.0209 (11)	0.0264 (12)	0.0254 (12)	0.0024 (9)	0.0041 (9)	0.0062 (10)
C12	0.0284 (13)	0.0333 (14)	0.0436 (16)	0.0016 (11)	0.0146 (12)	−0.0003 (12)
C13	0.0200 (11)	0.0226 (11)	0.0289 (12)	−0.0052 (9)	−0.0005 (9)	0.0041 (10)
C14	0.0197 (11)	0.0209 (11)	0.0220 (11)	−0.0013 (9)	−0.0012 (9)	−0.0009 (9)
C15	0.0169 (10)	0.0214 (11)	0.0161 (10)	−0.0022 (8)	−0.0013 (8)	−0.0020 (8)
C16	0.0213 (11)	0.0228 (11)	0.0172 (10)	−0.0007 (9)	−0.0009 (9)	−0.0010 (8)
C17	0.0297 (12)	0.0204 (11)	0.0236 (12)	−0.0003 (9)	−0.0027 (10)	−0.0011 (9)
C18	0.0270 (12)	0.0315 (13)	0.0239 (12)	0.0038 (10)	−0.0072 (10)	−0.0113 (10)
C19	0.0497 (17)	0.0370 (16)	0.0350 (15)	0.0097 (13)	−0.0074 (13)	−0.0184 (12)
C20	0.0285 (13)	0.0412 (15)	0.0183 (11)	0.0026 (11)	0.0011 (10)	−0.0072 (10)
C21	0.0251 (12)	0.0301 (13)	0.0193 (11)	−0.0016 (10)	0.0018 (9)	−0.0005 (10)

### Geometric parameters (Å, °)

**Table:**

Sn—O1	2.1590 (15)	C9—C10	1.380 (3)
Sn—O1^i^	2.1591 (15)	C9—H9A	0.9500
Sn—I1	2.76735 (17)	C10—C11	1.392 (3)
Sn—I1^i^	2.76737 (17)	C10—H10A	0.9500
Sn—I2^i^	2.81580 (19)	C11—C13	1.404 (4)
Sn—I2	2.8158 (2)	C11—C12	1.502 (3)
O1—P1	1.5207 (16)	C12—H12A	0.9800
P1—C15	1.786 (2)	C12—H12B	0.9800
P1—C8	1.791 (2)	C12—H12C	0.9800
P1—C1	1.796 (2)	C13—C14	1.385 (3)
C1—C7	1.389 (3)	C13—H13A	0.9500
C1—C2	1.396 (3)	C14—H14A	0.9500
C2—C3	1.382 (3)	C15—C16	1.391 (3)
C2—H2A	0.9500	C15—C21	1.405 (3)
C3—C4	1.393 (4)	C16—C17	1.387 (3)
C3—H3A	0.9500	C16—H16A	0.9500
C4—C6	1.396 (3)	C17—C18	1.394 (4)
C4—C5	1.503 (3)	C17—H17A	0.9500
C5—H5A	0.9800	C18—C20	1.387 (4)
C5—H5B	0.9800	C18—C19	1.502 (4)
C5—H5C	0.9800	C19—H19A	0.9800
C6—C7	1.395 (3)	C19—H19B	0.9800
C6—H6A	0.9500	C19—H19C	0.9800
C7—H7A	0.9500	C20—C21	1.384 (4)
C8—C9	1.394 (3)	C20—H20A	0.9500
C8—C14	1.402 (3)	C21—H21A	0.9500
			
O1—Sn—O1^i^	180.0	C9—C8—P1	117.67 (17)
O1—Sn—I1	89.84 (4)	C14—C8—P1	122.98 (17)
O1—Sn—I1	89.84 (4)	C10—C9—C8	120.5 (2)
O1^i^—Sn—I1	90.16 (4)	C10—C9—H9A	119.8
O1—Sn—I1^i^	90.16 (4)	C8—C9—H9A	119.8
O1^i^—Sn—I1^i^	89.84 (4)	C9—C10—C11	121.2 (2)
I1—Sn—I1^i^	180.0	C9—C10—H10A	119.4
O1—Sn—I2^i^	93.59 (4)	C11—C10—H10A	119.4
O1^i^—Sn—I2^i^	86.41 (4)	C10—C11—C13	118.4 (2)
I1—Sn—I2^i^	90.529 (6)	C10—C11—C12	120.2 (2)
I1^i^—Sn—I2^i^	89.471 (6)	C13—C11—C12	121.4 (2)
O1—Sn—I2	86.41 (4)	C11—C12—H12A	109.5
O1^i^—Sn—I2	93.59 (4)	C11—C12—H12B	109.5
I1—Sn—I2	89.469 (6)	H12A—C12—H12B	109.5
I1^i^—Sn—I2	90.531 (6)	C11—C12—H12C	109.5
I2^i^—Sn—I2	180.0	H12A—C12—H12C	109.5
P1—O1—Sn	149.47 (10)	H12B—C12—H12C	109.5
O1—P1—C15	114.91 (10)	C14—C13—C11	120.8 (2)
O1—P1—C8	109.87 (10)	C14—C13—H13A	119.6
C15—P1—C8	109.46 (11)	C11—C13—H13A	119.6
O1—P1—C1	108.25 (10)	C13—C14—C8	120.1 (2)
C15—P1—C1	106.96 (11)	C13—C14—H14A	120.0
C8—P1—C1	107.07 (10)	C8—C14—H14A	120.0
C7—C1—C2	119.4 (2)	C16—C15—C21	119.0 (2)
C7—C1—P1	120.92 (17)	C16—C15—P1	120.96 (17)
C2—C1—P1	119.66 (17)	C21—C15—P1	119.77 (18)
C3—C2—C1	120.4 (2)	C17—C16—C15	120.2 (2)
C3—C2—H2A	119.8	C17—C16—H16A	119.9
C1—C2—H2A	119.8	C15—C16—H16A	119.9
C2—C3—C4	120.9 (2)	C16—C17—C18	121.0 (2)
C2—C3—H3A	119.6	C16—C17—H17A	119.5
C4—C3—H3A	119.6	C18—C17—H17A	119.5
C3—C4—C6	118.5 (2)	C20—C18—C17	118.6 (2)
C3—C4—C5	120.1 (2)	C20—C18—C19	121.3 (2)
C6—C4—C5	121.4 (2)	C17—C18—C19	120.1 (3)
C4—C5—H5A	109.5	C18—C19—H19A	109.5
C4—C5—H5B	109.5	C18—C19—H19B	109.5
H5A—C5—H5B	109.5	H19A—C19—H19B	109.5
C4—C5—H5C	109.5	C18—C19—H19C	109.5
H5A—C5—H5C	109.5	H19A—C19—H19C	109.5
H5B—C5—H5C	109.5	H19B—C19—H19C	109.5
C7—C6—C4	120.9 (2)	C21—C20—C18	121.2 (2)
C7—C6—H6A	119.6	C21—C20—H20A	119.4
C4—C6—H6A	119.6	C18—C20—H20A	119.4
C1—C7—C6	119.9 (2)	C20—C21—C15	120.0 (2)
C1—C7—H7A	120.1	C20—C21—H21A	120.0
C6—C7—H7A	120.0	C15—C21—H21A	120.0
C9—C8—C14	119.1 (2)		
			
I1—Sn—O1—P1	60.78 (19)	C1—P1—C8—C14	84.5 (2)
I1^i^—Sn—O1—P1	−119.22 (19)	C14—C8—C9—C10	−0.6 (3)
I2^i^—Sn—O1—P1	−29.74 (19)	P1—C8—C9—C10	173.90 (18)
I2—Sn—O1—P1	150.26 (19)	C8—C9—C10—C11	0.0 (4)
Sn—O1—P1—C15	−31.2 (2)	C9—C10—C11—C13	0.7 (4)
Sn—O1—P1—C8	92.7 (2)	C9—C10—C11—C12	−177.7 (2)
Sn—O1—P1—C1	−150.70 (18)	C10—C11—C13—C14	−0.7 (4)
O1—P1—C1—C7	1.8 (2)	C12—C11—C13—C14	177.7 (2)
C15—P1—C1—C7	−122.5 (2)	C11—C13—C14—C8	0.1 (4)
C8—P1—C1—C7	120.2 (2)	C9—C8—C14—C13	0.5 (3)
O1—P1—C1—C2	−175.57 (19)	P1—C8—C14—C13	−173.65 (18)
C15—P1—C1—C2	60.1 (2)	O1—P1—C15—C16	86.7 (2)
C8—P1—C1—C2	−57.2 (2)	C8—P1—C15—C16	−37.4 (2)
C7—C1—C2—C3	−0.2 (4)	C1—P1—C15—C16	−153.10 (19)
P1—C1—C2—C3	177.2 (2)	O1—P1—C15—C21	−87.5 (2)
C1—C2—C3—C4	−1.1 (4)	C8—P1—C15—C21	148.36 (18)
C2—C3—C4—C6	2.2 (4)	C1—P1—C15—C21	32.7 (2)
C2—C3—C4—C5	−177.3 (2)	C21—C15—C16—C17	0.1 (3)
C3—C4—C6—C7	−2.2 (4)	P1—C15—C16—C17	−174.16 (18)
C5—C4—C6—C7	177.4 (2)	C15—C16—C17—C18	1.1 (4)
C2—C1—C7—C6	0.3 (4)	C16—C17—C18—C20	−1.4 (4)
P1—C1—C7—C6	−177.14 (19)	C16—C17—C18—C19	179.9 (2)
C4—C6—C7—C1	0.9 (4)	C17—C18—C20—C21	0.6 (4)
O1—P1—C8—C9	27.6 (2)	C19—C18—C20—C21	179.3 (3)
C15—P1—C8—C9	154.69 (18)	C18—C20—C21—C15	0.5 (4)
C1—P1—C8—C9	−89.72 (19)	C16—C15—C21—C20	−0.9 (3)
O1—P1—C8—C14	−158.13 (18)	P1—C15—C21—C20	173.47 (19)
C15—P1—C8—C14	−31.1 (2)		

Symmetry codes: (i) −*x*, −*y*+1, −*z*+1.
